# Methotrexate‐Loaded Liposomal Formulation Enables 6‐Week Sustained Intraocular Therapeutic Drug Release in a Porcine Model

**DOI:** 10.1002/adhm.202503230

**Published:** 2025-09-25

**Authors:** Maximilian Hammer, Lea Skrzypczyk, Sabrina Wohlfart, Bryan Calder Ackermann, Ludwig Geisweid, Simon William Pohl, Margarita Karaivanova, Jonathan Herth, Victor Aristide Augustin, Alexander Studier‐Fischer, Tina Sackmann, Leon Kaulen, Anna Steyer, Walter Mier, David H Steel, Kanmin Xue, Gerd Uwe Auffarth, Philipp Uhl

**Affiliations:** ^1^ David J Apple Laboratory for Vision Research and University Eye Clinic Heidelberg 69120 Heidelberg Germany; ^2^ Faculty of Biosciences Heidelberg University 69120 Heidelberg Germany; ^3^ Department of Nuclear Medicine Heidelberg University Hospital 69120 Heidelberg Germany; ^4^ Institute of Pharmacy and Molecular Biotechnology 69120 Heidelberg Germany; ^5^ Department of Urology and Urological Surgery University Medical Center Mannheim 68167 Mannheim Germany; ^6^ Institute of Physiology and Pathophysiology Heidelberg University Im Neuenheimer Feld 326 69120 Heidelberg Germany; ^7^ Department of Neurology Division of Neuro‐Oncology Massachusetts General Hospital Harvard Medical School Boston MA MA 02114 USA; ^8^ Department of Neurology University Hospital Heidelberg Heidelberg University 69120 Heidelberg Germany; ^9^ EMBL Imaging Centre European Molecular Biology Laboratory 68167 Heidelberg Germany; ^10^ Bioscience Institute Newcastle University, Newcastle Upon Tyne UK and Sunderland Eye Infirmary Queen Alexandra Road Sunderland SR2 9HP UK; ^11^ Nuffield Laboratory of Ophthalmology Nuffield Department of Clinical Neurosciences University of Oxford Oxford OX3 9DU UK; ^12^ Oxford Eye Hospital Oxford University Hospitals NHS Foundation Trust Oxford OX3 9DU UK

**Keywords:** intravitreal injection, liposomes, methotrexate, nanocarrier, sustained drug release

## Abstract

Methotrexate (MTX) inhibits cell proliferation, which underlies ocular diseases, including intraocular lymphoma and proliferative vitreoretinopathy. However, MTX normally requires bi‐weekly intravitreal injections due to a short half‐life, causing rapid clearance below therapeutic thresholds within 72h. To overcome these limitations, sustained‐release carriers, including poly(lactic‐co‐glycolic) acid‐based implants, were investigated in vitro previously. These systems offer prolonged drug delivery but require relatively large‐gauge surgical implantation, which increases the risk of surgical complications and limits their practical use. In this study, MTX‐loaded liposomes that can be administered via a 30‐gauge cannula are developed, obviating the need for more invasive surgical implantation. This phospholipid‐based liposomal formulation succeeded in enabling sustained methotrexate release at therapeutic levels for over six weeks following a single intravitreal injection, demonstrated in vivo in a large animal pig model. High biocompatibility of this novel liposomal formulation is confirmed through longitudinal retinal structure (optical coherence tomography) and function (electroretinography) assessments. This liposomal formulation of MTX provides a clinically and surgically optimized drug delivery system that allows improved management of intraocular lymphoma and proliferative vitreoretinopathy in the future.

## Graphical abstract

A liposomal formulation for methotrexate enables minimally invasive, sustained intraocular drug delivery for over six weeks with a single injection. Developed for treating intraocular diseases like lymphoma and PVR, it offers excellent biocompatibility in vivo in a porcine model. This innovative approach is non‐inferior to repetitive bi‐weekly injections of free methotrexate, providing a safer, and cost‐effective alternative.

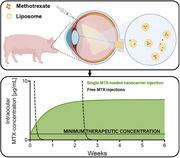

## 1 Introduction

Intravitreal drug administration has revolutionized the treatment of retinal diseases. By providing direct access to the retina, systemic side effects are minimized. However, the half‐life and, therefore, the needed injection frequency strongly varies between protein, peptide, and small‐molecule drugs. While the vitreous body, a gel‐like substance that fills most of the eye's volume, acts as a natural drug reservoir for protein compounds,^[^ ^1^, ^2^ ^]^ the half‐life of small molecule drugs is, in most cases, extremely short, necessitating frequent application.^[^ ^3^ ^]^ Thus, while efficacy might be proven, the clinical applicability of intravitreally applied small‐molecule drugs is limited. Among these is methotrexate (MTX), a small‐molecule folic acid antimetabolite.^[^ ^4^ ^]^ MTX has been proven to be effective in the treatment of intraocular lymphoma^[^ ^5^, ^6^ ^]^ and recently also in the treatment of proliferative vitreoretinopathy (PVR), a postoperative progressive scarring of the retina, as the first effective pharmacological intervention.^[^ ^7^ ^]^

Despite these promising results, methotrexate has not reached widespread clinical use due to the short intravitreal half‐life of roughly 7.6 h.^[^ ^8^, ^9^ ^]^ This translates to only 72 h of therapeutic levels (0.1–2 µg mL^−1^) after an intravitreal injection of 400 µg, requiring a typical bi‐weekly injection regime in the treatment of lymphoma.^[^ ^5^ ^]^ To date, no sustained release carriers are available, and the high MTX concentration spikes (up to 600 µg mL^−1^ or 1321 µm) after injections with possible cytotoxic effects and complications, including endophthalmitis and corneal epitheliopathy occurring in nearly a quarter of patients^[^ ^10^ ^]^ are accepted. These downsides, together with the significant financial, psychological, and social burden on the patients’ life, prevent the widespread use of MTX for other indications.^[^ ^11^ ^]^

These major limitations could be overcome by sustained drug delivery systems. One approach to sustain the release of methotrexate is by using poly(lactic‐co‐glycolic) (PLGA)‐based solid implants previously only evaluated in vitro,^[^ ^12^ ^]^ similar to the approved PLGA‐implant of dexamethasone marketed as “Ozurdex” (Allergan, Irvine, USA).^[^ ^13^ ^]^ While some limitations, especially the treatment burden and high peak concentrations, can be eliminated by this approach, due to the dimension of the implants, expensive manufacturing and specific relatively large gauge (22‐gauge) injectors are required.^[^ ^14^ ^]^ This could be a concern, especially in cases with intraocular lymphoma due to a higher risk of tumor seeding.

Among other nanocarriers such as nanogels, dendrimers, and Poly(lactic acid) nanoparticles,^[^ ^15^, ^16^ ^]^ phospholipid‐based liposomal formulations creating a second diffusion barrier for small molecules represent a promising alternative for slow‐release intravitreal drug delivery.^[^ ^17^ ^]^ The liposomes’ small size of 100–150 nm^[^ ^18^ ^]^ and their structure do not impair visual performance while still enabling controlled drug release, significantly extending the drug's half‐life in the vitreous,^[^ ^19^ ^]^ and in some cases allowing improved biocompatibility.^[^ ^20^ ^]^ Furthermore, narrow gauge 30G needles can be used to inject liposomal formulations not requiring sophisticated injector systems, and dosing can be titrated easily via the injected volume for different indications, compared to solid implants, providing a cost‐effective alternative.

**Figure 1:**
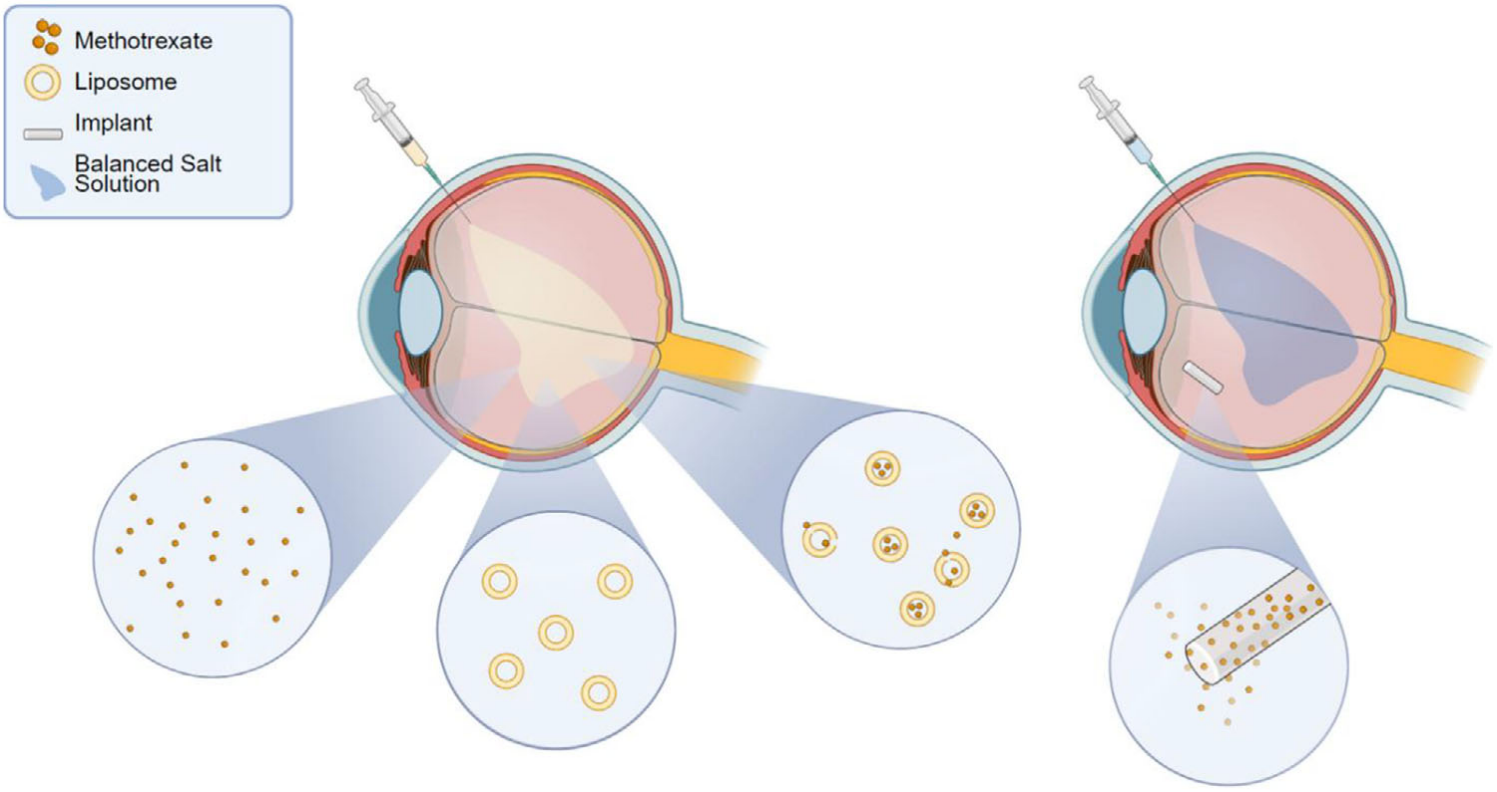
In vivo evaluation of a liposomal methotrexate (MTX) sustained drug release system in the porcine model. Free MTX (n=1), empty (n=3) and MTX‐loaded, phospholipid‐based liposomes (n=7), as well as an MTX‐loaded poly(lactic‐co‐glycolic) acid (PLGA) implant (n=2) and balanced salt solution (BSS) (n=3) were administered by intravitreal injections to assess their pharmacokinetics and biocompatibility in the eyes of pigs.

**Figure 2:**
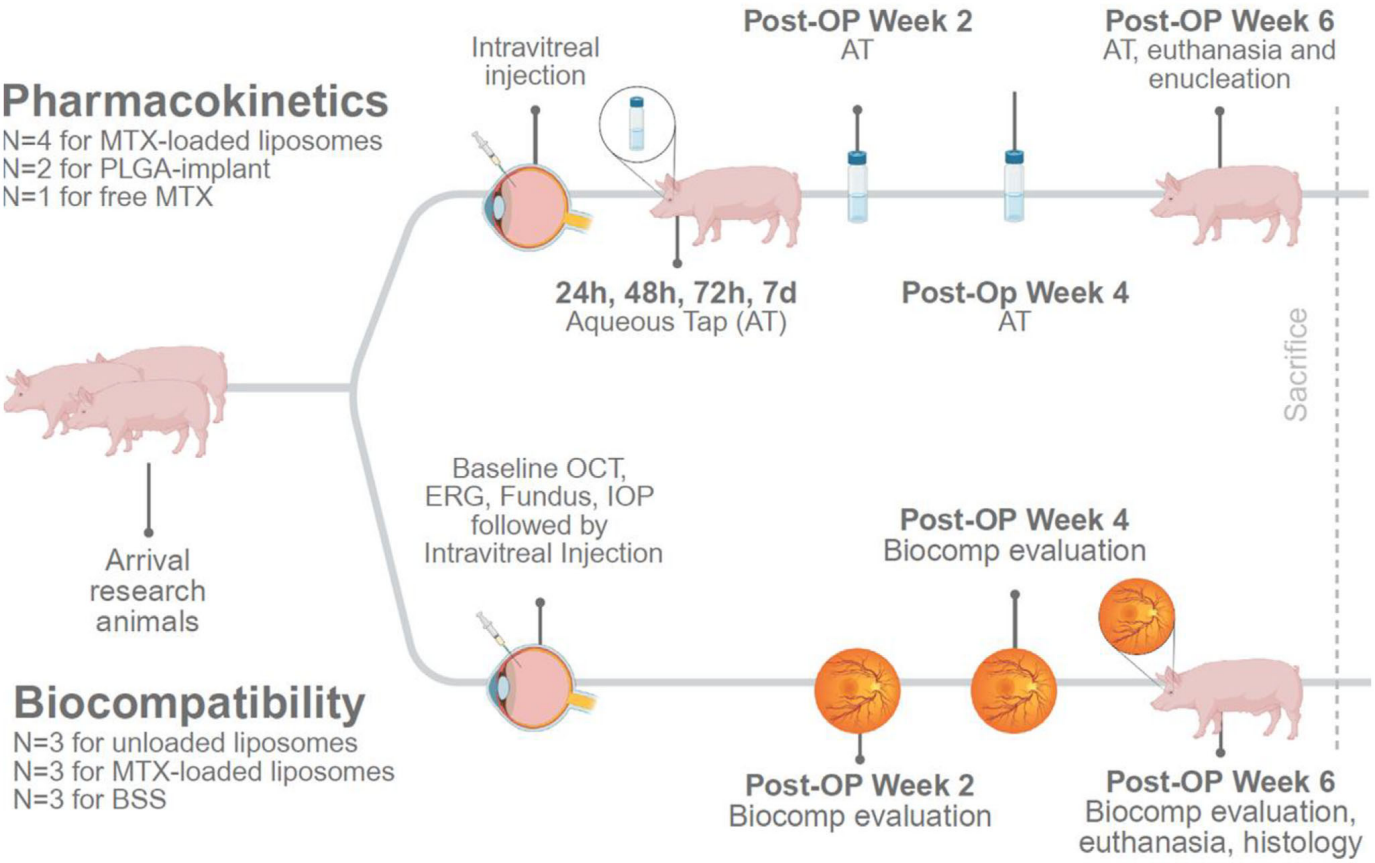
Study protocol. 16 pigs were divided into two cohorts for the assessment of i) pharmacokinetics (in total *n* = 7: *n* = 4 for liposomes, *n* = 2 for poly(lactic‐co‐glycolic) acid (PLGA)‐implant, *n* = 1 for free methotrexate (MTX)) and ii) biocompatibility (*n* = 6) of the liposomal formulation compared to balanced salt solution (BSS) (*n* = 3). Intraocular MTX levels were determined by aqueous taps from the anterior chamber on days 1, 2, and 3, as well as after 1, 2, 4, and 6 weeks. Biocompatibility was assessed 1, 2, 4, and 6 weeks after the initial intravitreal injection. Two additional animals underwent implantation of the PLGA‐based implant.

Both approaches, liposomal formulations of methotrexate and solid implants made from PLGA, have never been compared in vivo (**Figure** **1**). More specifically, in contrast to the topical use of liposomal formulations on the cornea, the intraocular pharmacokinetics of intravitreally injected liposomal formulations with current standards (very low polydispersity index and a diameter of 100–120 nm) had never been investigated in a large animal model. Further, biocompatibility assessments of these formulations had never been performed in a translational large animal model. The primary aim of this study was therefore to evaluate the liposomal pharmacokinetics in vivo and to directly compare them to more invasive delivery methods like PLGA‐implants. We chose MTX‐loaded liposomes as there is a high clinical need for sustained drug release systems, particularly for small‐molecule and antiproliferative drugs. Detailed ocular assessments including fundus photography, intraocular pressure measurements, electroretinography (ERG) and optical coherence tomography (OCT) were performed throughout the study to evaluate biocompatibility of the phospholipid‐based liposomes as well as for liposomes loaded with methotrexate (**Figure** **2**). We hypothesized that by characterizing liposomal formulations to extend the therapeutic effect of methotrexate, the number of required injections can be reduced, thereby lowering the patient's treatment burden. This enables MTX to be a sustainable, resource‐ and cost‐efficient clinically applicable treatment option.

## 2 Results

### 2.1 Characterization of Liposomes

**Figure 3:**
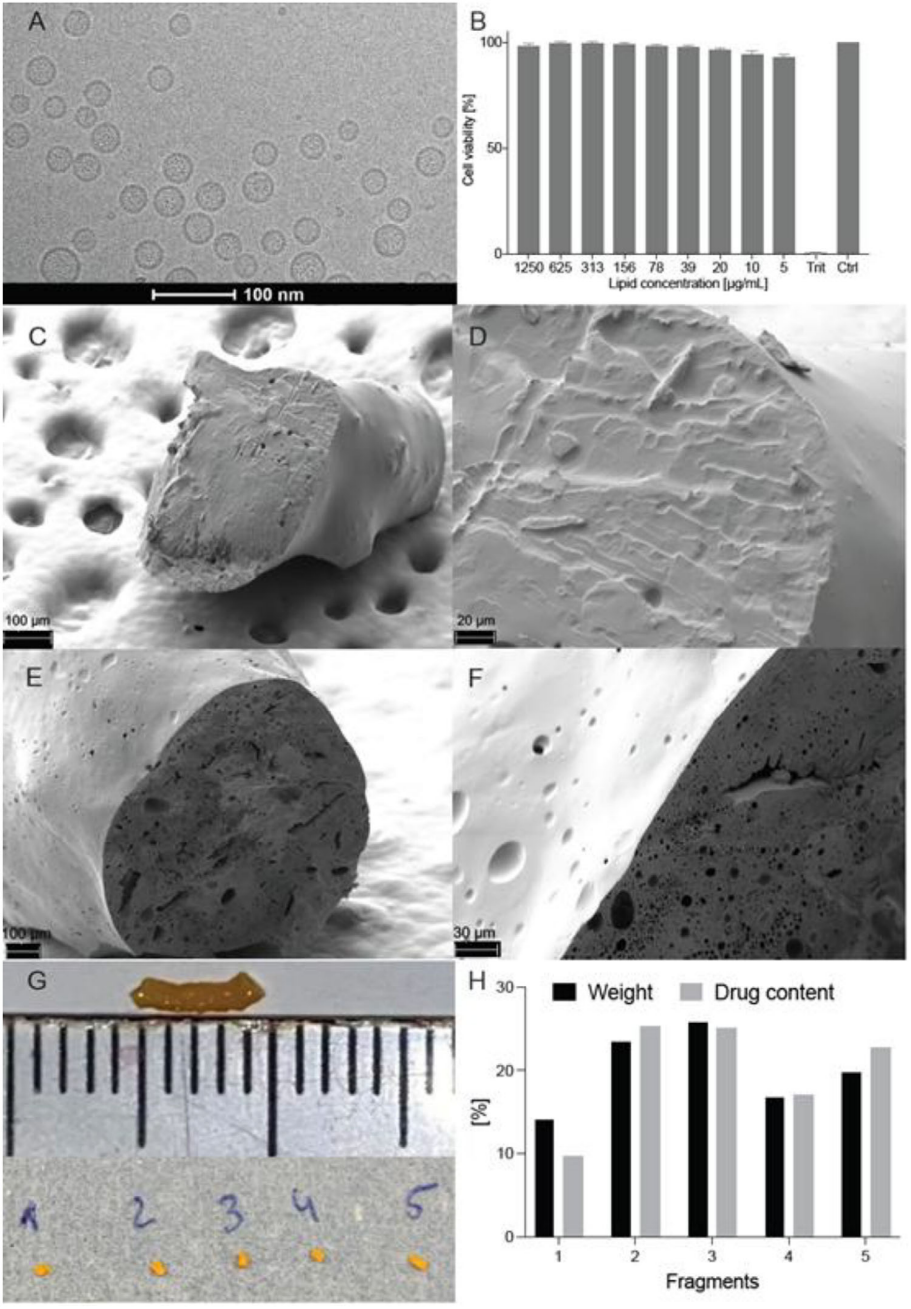
Characterization of methotrexate (MTX)‐loaded liposomes and poly(lactic‐co‐glycolic) acid (PLGA) implant. A) MTX‐loaded liposomes were generally uniform in size and showed unilamellar structure in cryo‐Transmission Electron Microscopy (cryo‐TEM). B) Consistently high levels of in vitro biocompatibility were observed for the unloaded liposomal formulation in ARPE‐19 cells (a human retinal pigment epithelium cell line, *n* = 3). The control group does not showcase an error bar as data is normalized to 100% in every run. C,D) Prior to examination in the posterior segment perfusion model, an MTX‐loaded PLGA implant showed a smooth surface and solid inner structure as visualized using scanning electron microscopy. E,F) After completing 21 days in an in vitro posterior segment eye model, the carrier turned porous. G) The PLGA‐implant was divided into 5 different segments to assess how homogenously MTX is located within the implant. H) A very homogenous MTX‐distribution between the single segments was observed by quantification of MTX content via reverse‐phase High‐performance liquid chromatography (HPLC).

Liposomal formulations with BSS or with MTX dissolved in BSS were characterized by dynamic light scattering in terms of size, polydispersity index (PDI), and zeta potential. The average size of the liposomes containing BSS only was 115.6 nm (SD = ± 0.43) with a PDI of 0.242 (SD ± 0.034) and a zeta potential of −10.75 mV. The average size of liposomes obtained with MTX solution was 119.5 nm (SD = ± 0.45) with a PDI of 0.197 (SD = ± 0.009) and zeta potential of ‐10.66 mV. A high encapsulation efficiency of 57.13% with a drug loading of ≈14.44% could be achieved as determined by reverse‐phase High‐performance liquid chromatography (HPLC). A respective cryo‐Transmission Electron Microscopy (cryo‐TEM) image of the MTX‐loaded formulation is presented in **Figure** **3**A, demonstrating low lamellarity (mainly unilamellarity) of the liposomes.

### 2.2 In Vitro Cytotoxicity Assessment

The viability of human Retinal Pigment Epithelial (RPE) cells (ARPE‐19) was not impaired by the addition of unloaded liposomal formulation in a concentration range from 0.005 to 1.25 mg mL^−1^, as depicted in Figure 3B.

### 2.3 Characterization of Degradation of PLGA Implants

Prior to the evaluation of the PLGA implants in the porcine model, the degradation was investigated in an in vitro posterior segment perfusion model. Cryo‐SEM images prior to and after 21 days in the perfusion models were recorded and are presented in Figure 3C–F. Further, to showcase the homogenous MTX‐distribution in the implant, we divided the implants into 5 parts (Figure 3G) and evaluated MTX content per weight. A very homogeneous MTX distribution was observed. (weight: drug ratio 1.05 ± 0.1 mean ± SEM, Figure 3H).

### 2.4 In Vitro and In Vivo Methotrexate Release From MTX‐Loaded Liposomes and PLGA Formulations in an In Vitro Posterior Segment Perfusion Model and in the Porcine Model

For in vivo pig experiments, the animals were divided into four groups (**Table** **1**).

**Table 1:** Overview of the in vivo experiments in pigs.

Injected formulation	Biocompatibility	Pharmacokinetics	Dosage
MTX‐liposomes	*n* = 3	*n* = 4	400 µg MTX / 0.075 mL
Unloaded Liposomes	*n* = 3	n/a	Same lipid concentration as MTX‐liposomes / 0.075 mL
Free MTX	n/a	*n* = 1	400 µg MTX / 0.1 mL
SHAM Injection (Balanced Salt Solution)	*n* = 3	n/a	0.1 mL of BSS

**Figure 4:**
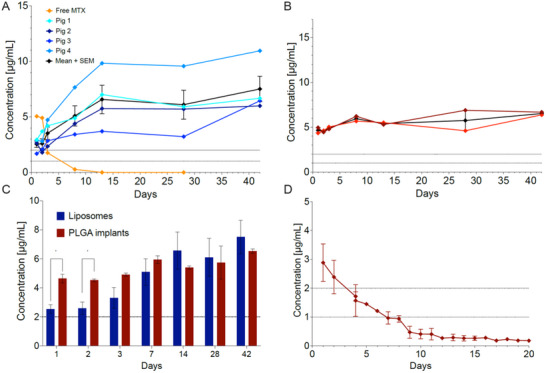
Pharmacokinetics of methotrexate release in porcine eyes following intravitreal administration. A) Methotrexate (MTX) concentrations in the aqueous humor following intravitreal injection of 400 µg of free MTX (orange line, *n* = 1) or 400 µg of liposomal MTX (*n* = 4, each colored line representing an individual eye, black line represents mean ± SEM). B) MTX concentrations in the aqueous humor following intravitreal injection of a solid PLGA implant containing 400 µg of MTX (*n* = 2, each colored line representing an individual eye, black line represents mean ± SEM). C) After a two‐day ramping phase, no significant difference in aqueous MTX concentration was detected between eyes injected with the MTX‐liposomes (*n* = 4) versus PLGA‐formulations (*n* = 2) of MTX. ^*^ *p* < 0.05, multiple unpaired *t*‐tests. D) In vitro MTX release from solid PLGA implant with a simulated aqueous flow rate of 2.5 µL min^−1^ (*n* = 3, mean ± SEM is presented).

One animal injected with 400 µg of free MTX showed the expected exponential decay falling below therapeutic concentrations within 5 days (**Figure** **4**A). Following intravitreal injection of 400 µg of MTX‐liposomes in 0.075 mL, drug release was monitored over a period of 6 weeks in four pigs. The release profile shows that the methotrexate concentration in the aqueous humor gradually rose until 14 days with a *Cmax* of 6.6 ± 1.3 µg mL^−1^ (Figure 4A). Subsequently, MTX concentration remained above the therapeutic concentration of 1 µg mL^−1^ throughout the 6‐week observation period.^[^ ^21^ ^]^ At the end of the study (day 42), the mean concentration was 7.5 ± 1.2 µg mL^−1^. These results demonstrate that a single intravitreal injection of the MTX‐loaded liposomal formulation can provide sustained drug release and maintain therapeutically relevant MTX concentrations in the aqueous humor of eyes similar in size to humans over an extended period of time.

As a comparison, in vivo release of MTX from PLGA‐based rods in porcine eyes also demonstrated sustained drug release over 42 days (Figure 4B). Initial MTX concentrations on day 1 were ≈4–5 µg mL^−1^ in both pigs (Figure 4B). A stable release phase was observed between days 2 and 14, with concentrations ranging from ≈4.5 to 6 µg mL^−1^ in both animals (Figure 4B) up to day 42. Throughout the study, MTX levels remained above the therapeutic threshold of 1–2 µg mL^−1^. A direct comparison between the MTX‐liposomes and the PLGA‐implant is presented in Figure 4C. Single data points for all animals undergoing pharmacokinetic evaluation are provided in Table S1 (Supporting Information). For the same PLGA‐implant studied in the posterior segment model with a simulated aqueous flow rate of 2.5 µL min^−1^, a steady state MTX concentration of mean 0.3 ± 0.1 µg mL^−1^ (SEM) was achieved (Figure 4D).

### 2.5 Biocompatibility Evaluation of Intravitreally Administered Methotrexate‐Loaded and Control Liposomal Formulations

**Figure 5:**
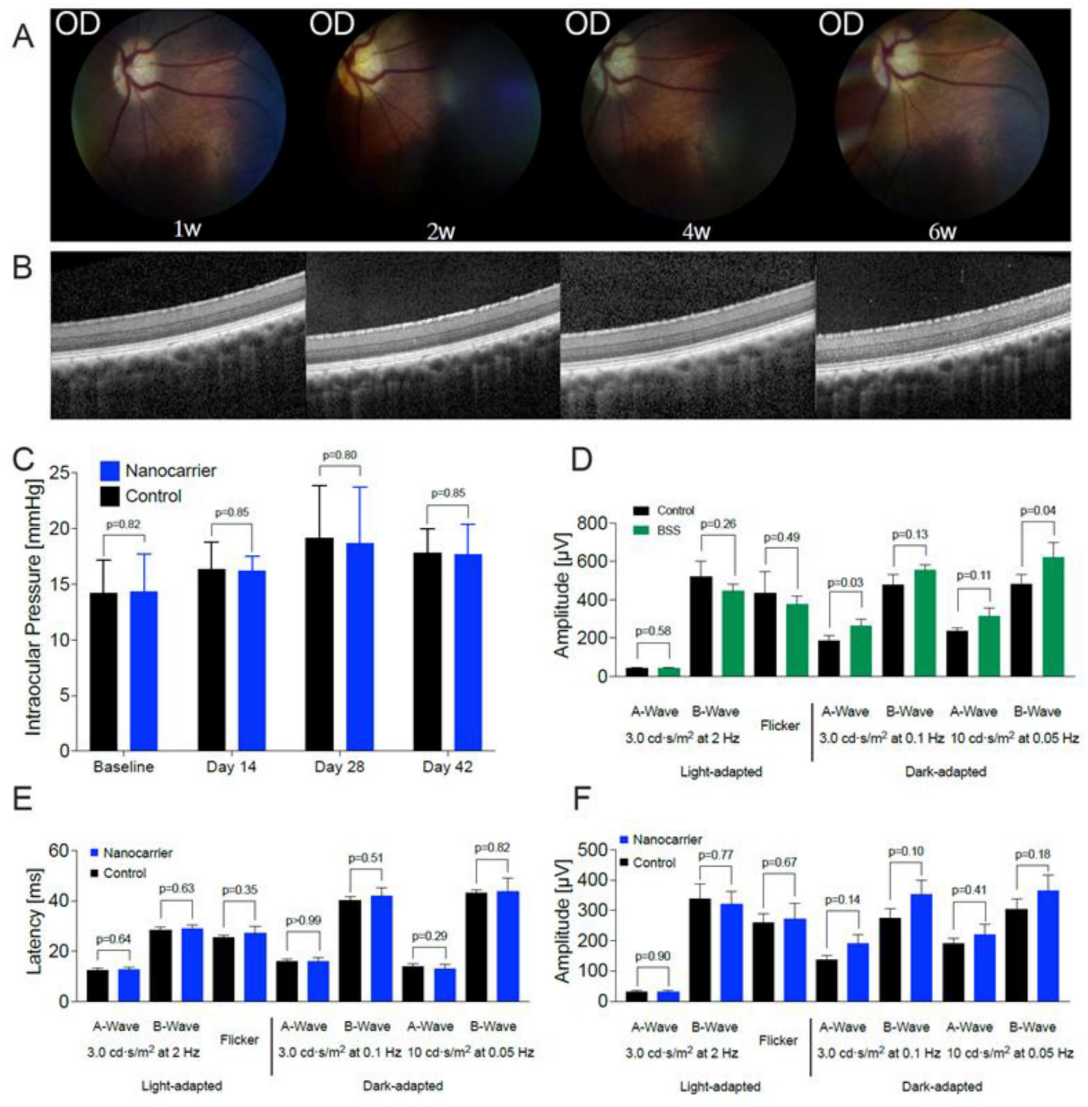
In vivo biocompatibility assessment of methotrexate (MTX)‐liposomes. The MTX‐loaded (*n* = 3) or unloaded (*n* = 3) liposomes were administered by intravitreal injection, followed by a follow‐up of 6 weeks with longitudinal fundal imaging, Optical Coherence Tomography (OCT), and histological analysis. A) Representative color fundus photographs from one eye from 1, 2, 4, and 6 weeks after treatment with the MTX‐liposomes showing no significant retinal changes over time. B) Corresponding OCTs from the same timepoints showing no significant change in retinal structure over time. In particular, no signs of cystoid edema or retinal atrophy were observed. C) Intraocular pressure was within normal limits throughout the experiment in all study and control eyes with no significant difference between the two groups (multiple paired *t*‐tests (fellow eyes paired), *n* = 6 in total, *n* = 3 for unloaded and MTX‐loaded liposomes, respectively). D) Comparison of animals treated with Balanced Salt Solution (BSS) (*n* = 3, multiple paired *t*‐tests (fellow eyes paired)) showed similar non‐clinically relevant differences between injected and control eyes at 6 weeks similar to no clinically relevant changes in latency E) and amplitudes F) of animals injected with liposomal formulations (*n* = 6, *n* = 3 unloaded liposomes, *n* = 3 MTX‐loaded liposomes) (multiple paired *t*‐tests (fellow eyes paired)).

**Figure 6:**
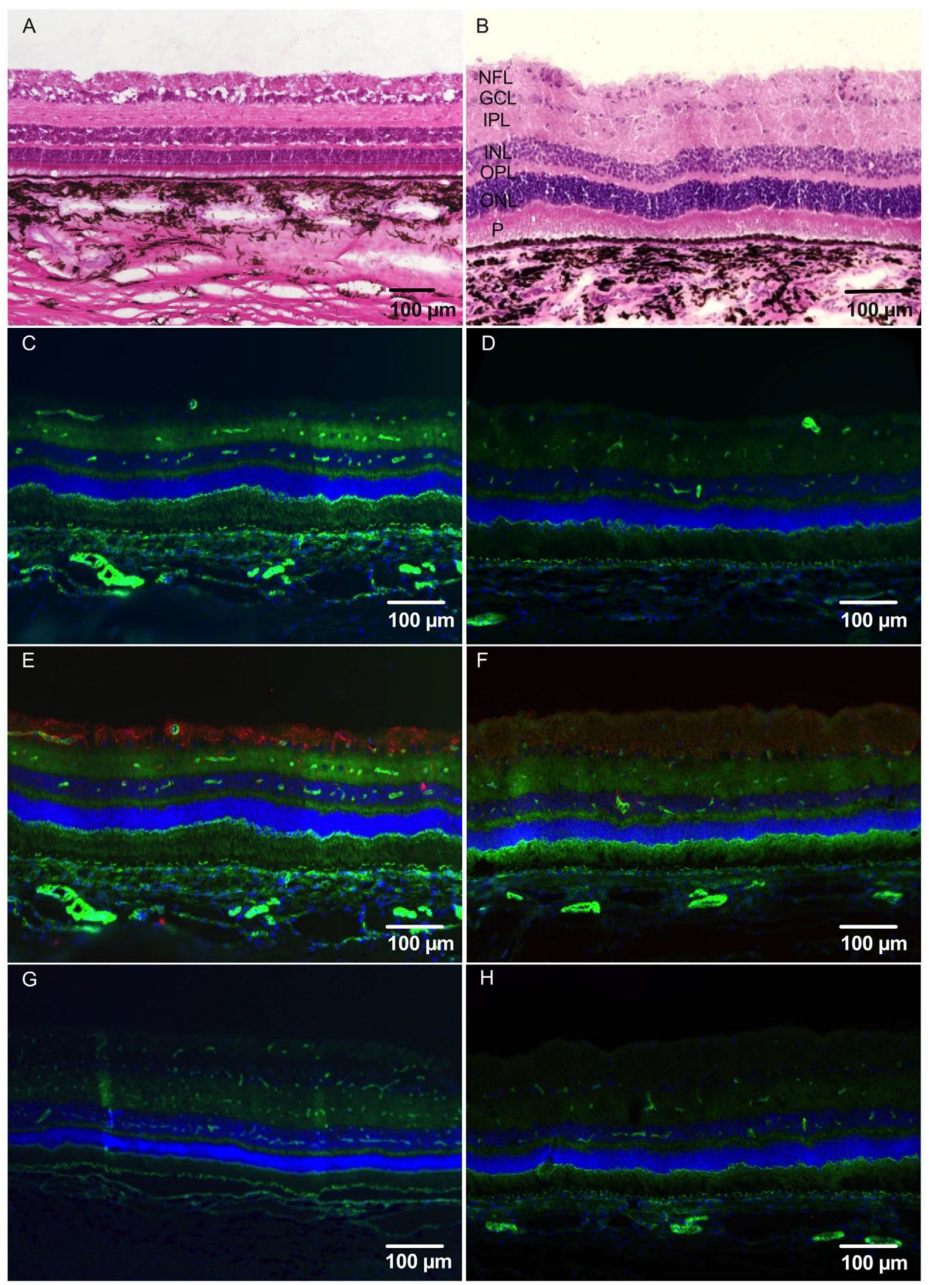
Histological and immunohistochemical analysis of the retina following intravitreal injection of unloaded or methotrexate (MTX)‐loaded liposomes and control eyes. A,B) Hematoxylin and eosin (H&E) staining of retinal sections. A) Retina from an eye injected with unloaded liposomes and MTX‐loaded B) liposomes. No loss of cell density is visible even at higher magnification. C–H) Immunohistochemical staining of inflammatory and glial markers. Control eyes (left eyes, no injections) are shown in C, E, and G; MTX‐loaded liposome‐treated eyes (right eyes) are shown in D, F, and H. C,D) CD45 staining (red) for leukocyte infiltration, E,F) GFAP staining (red) for Müller cell activation, and G,H) Iba1 staining (red) for microglia. Phalloidin (green) was used to visualize the actin cytoskeleton, and DAPI (blue) was used for nuclear staining in all panels. CD45 and Iba1 staining revealed no appreciable immune cell infiltration or microglial activation in either group. GFAP shows a typical distribution in the inner retina. Abbreviations: NFL – nerve fibre layer, GCL – Ganglion Cell Layer, IPL‐ Inner Plexiform Layer, INL – Inner Nuclear Layer, OPL – Outer Plexiform Layer, ONL – Outer Nuclear Layer, P = Photoreceptor Layers.

The biocompatibility of unloaded and MTX‐loaded liposomes was evaluated in six animals (*n* = 3 each) over 6 weeks. Fundoscopy showed normal retinal and vascular appearance with no evidence of intraocular inflammation (**Figure** **5**A). Optical Coherence Tomography (OCT) imaging showed preserved retinal layers with no evidence of retinal oedema, atrophy, detachment or other structural abnormalities (Figure 5B). The mean central retinal thickness remained comparable before and after treatment (mean 278 ± 13 and 289 ± 18 µm (SEM) at baseline and 6 weeks, respectively, *p* = 0.14, paired *t*‐test). Intraocular pressures (IOP) remained within normal limits at all time points (Figure 5C). Animals that underwent SHAM‐injections with balanced salt solution showed similar biocompatibility results with electroretinography (ERG) results presented in Figure 5D. ERG in the 6 eyes injected with liposomal formulations showed no significant intra‐individual difference in a‐ and b‐wave amplitude or latency at six weeks compared to the contralateral control eye (Figure 5E,F). Histological analysis showed no disruption of retinal architecture in Hematoxylin and Eosin (H&E) staining of eyes injected with unloaded liposomal formulations (**Figure** **6**A) or MTX‐loaded liposomes (Figure 6B). Immunostainings of eyes injected with MTX‐liposomes showed no increased leukocyte infiltration, no increase in glial proliferation and no increased microglia activation compared to untreated left control eyes (Figure 6C–H).

## 3 Discussion

In this study, we present a novel strategy using a liposome optimized for the intravitreal application of methotrexate. The formulation had high biocompatibility and sustained release pharmacokinetics in a porcine model over 6 weeks, which represents a major increase in duration compared to free MTX administered intravitreally. Furthermore, the liposomal formulation was compared to a solid PLGA‐implant that would need to be surgically implanted through a larger incision and proven to be similar in pharmacokinetics while injectable through a standard 30‐gauge needle. Further, all components of the presented formulation are readily available in Good Manufacturing Practice (GMP)‐quality. With these advantages in mind, our study is a step forward toward clinical evaluation of MTX‐loaded liposomal formulations in Primary Vitreoretinal Lymphoma in the future, after ensuring Good Laboratory Practice (GLP)‐level toxicity studies are in place.

Previously, liposomal formulations for intravitreal drug delivery have been investigated for several drugs.^[^ ^19^ ^]^ While also shown to increase the intraocular levels of large molecular size antibody‐based therapies,^[^ ^22^ ^]^ the greatest effects were observed for small‐molecule drugs that are generally quickly washed out from the vitreous cavity. These studies focused mainly on drugs for treating infections with liposomal formulations developed and tested in various animal models for antibiotic,^[^ ^23^, ^24^, ^25^, ^26^ ^]^ antiviral,^[^ ^27^ ^]^ and antifungal^[^ ^28^ ^]^ drugs. To date, no study has examined the pharmacokinetics of intravitreally applied MTX‐loaded liposomes. Regarding antiproliferative agents for retinal application, previous studies investigated the intravitreal application of 5′‐fluorouracil (5‐FU) and its derivatives. However, only larger carriers in the µm‐range were used, and the authors do not report drug loading or encapsulation efficiency, making a direct comparison of results difficult.^[^ ^29^, ^30^, ^31^, ^32^ ^]^ For doxorubicin, a commercially available liposomal formulation (Lipo‐dox, Tung Yang Chemical Industries Co., Taiwan) has been evaluated after injection into the vitreous cavity of rabbits, but the authors did not report the applied dosage or any specifications of the used formulations. Moreover, doxorubicin was not detectable after 7 days, even when formulated in liposomes.^[^ ^33^ ^]^ In contrast, we developed a liposomal formulation for MTX and detected therapeutic levels (> 8 µg mL^−1^) in aqueous samples for 6 weeks after intravitreal injection. Similar concentrations of MTX have been previously shown to effectively inhibit proliferation whilst not inducing apoptosis or reducing cell viability in vitro when tested on immortalized photoreceptor and RPE cell lines.^[^ ^34^ ^]^ Further, exploratory experiments revealed no unwanted interaction of the liposomes with silicone oil endotamponades that may be used during vitreoretinal surgery (Figure S1, Supporting Information).

As no direct comparison to previously reported liposomal formulations was possible, in this study, the MTX‐liposomes were compared to a solid PLGA‐implant. This implant was chosen as it closely resembles a PLGA‐matrix already used the clinically‐approved dexamethasone implant, Ozurdex (Allergan, Irvine, USA).^[^ ^14^ ^]^ It was preferred over other solid implants previously tested in rabbit models containing chitosan,^[^ ^35^ ^]^ because it is not yet approved for intraocular use by licensing authorities. Between the two formulations, aqueous MTX concentrations were not significantly different after a ramping phase of 2 days. One major limitation of previously tested intravitreally delivered liposomal formulations was the high variability of drug levels.^[^ ^28^ ^]^ This was not observed for our MTX‐liposomal formulation, which performed equally well as the solid PLGA implant. In addition, the MTX‐liposome demonstrated good biocompatibility, with no associated retinal toxicity observed during in vivo testing in a large animal model with eye volumes similar to the human. In fact, in an early pioneering study conducted by Liu et al,^[^ ^36^ ^]^ liposomal formulations of the antiproliferative drug cytarabine majorly improved biocompatibility compared to the free drug, further supporting the approach presented in the present study. We believe that three important factors might explain the increased sustained release for MTX presented here in comparison to previous liposomal formulations tested intravitreally. First, previous liposomal formulations tested have shown particle sizes in the µm‐range rather than the nm range presented here and do not report on lamellarity, most likely leading to unstable release kinetics with occurring burst‐release. Further, previous studies evaluated drug levels after euthanasia and the removal of the vitreous body, while in the present study, repetitive aqueous humor taps were conducted to allow the evaluation of intraindividual pharmacokinetics. Next to majorly reducing animal numbers, this approach allowed to immediately measure samples without any denaturalization minimizing the risk of breakdown the liposomal formulation artificially increased MTX‐concentrations. Utilizing aqueous samples to detect the efficacy in intravitreally injected drugs to lower cytokines is accepted and often applied in clinical studies.^[^ ^37^ ^]^ Further, an animal injected with free MTX showed a similar exponential decrease in MTX‐levels as previously reported in studies utilizing a vitreous body in 21 rabbits.^[^ ^8^ ^]^ Lastly, the currently clinically applied dosage of 400 µg of MTX is an extremely high dose that cannot be applied, e.g., for other tested substances like voriconazole^[^ ^3^ ^]^ due to toxicity reasons, effectively limiting the release due to applied dosage.

Intravitreal delivery of the PLGA‐based Ozurdex implant requires a specialized 22‐gauge injector device, which can be associated with significant patient discomfort.^[^ ^38^ ^]^ Compared with solid MTX implants, the application of liposomal MTX could be easily performed through small‐gauge (30G) intravitreal injection needles, which are available in all ophthalmic practices and provide the flexibility to adjust the dosage by varying the injection volume. In addition, for the prevention of proliferative vitreoretinopathy, our formulation could potentially be given as an adjunct at the end of standard vitrectomy surgery for retinal detachment repair through existing 25G or 27G trocars and be compatible with gas and silicone oil endotamponades as demonstrated in pilot experiments (Figure S1, Supporting Information).

We also evaluated MTX release from the PLGA implants in vitro, which enables correlation between in vivo and in vitro observations. The applied syringe pump model is a frequently used in vitro model to simulate aqueous flow, not only for posterior segment pharmacokinetics^[^ ^12^ ^]^ but also for studies of the trabecular meshwork outflow.^[^ ^39^ ^]^ The results obtained suggest that in vivo testing is still required. The comparison of additional novel formulations using a combination of high‐throughput in vitro models and more limited in vivo validation may help to further elucidate their correlation.

### 3.1 Limitations

Due to the use of landrace pigs and their rapid growth curve, the follow‐up duration after intravitreal injection was limited to 6 weeks. However, the ocular anatomy of landrace pigs closely resembles that of the human eye, including a cone‐enriched central area of retina (similar to the macula in humans) and a trabecular meshwork outflow system. In addition, 6 weeks of follow‐up is a clinically relevant postoperative time period for the development of PVR as well as a feasible retreatment interval. In the present study, we successfully evaluated pharmacokinetics, thus, in further studies, efficacy in PVR or intravitreal lymphoma still needs to be proven. Further, while we detected homogenous MTX‐distribution in the PLGA‐implants, MTX‐loaded PLGA implants prepared using the hot‐melt extrusion method in a more controlled fashion should be evaluated in the future in case of aiming for clinical translation.

## 4 Conclusion

In summary, we present a novel strategy using a liposomal formulation for sustained intravitreal release of the anti‐proliferative agent, methotrexate, over 6 weeks. While no pharmacodynamic evaluation was conducted, its favorable pharmacokinetics with low inter‐individual variability and high biocompatibility as determined by in vivo testing, would support clinical investigation in a future clinical trial.

## 5 Experimental Section

### Materials

A full list of all materials is presented in the Supporting Information.

### Liposomal preparation

Liposomes were prepared by the thin film method and dual centrifugation using a Zentrimix 380 R (Hettich GmbH & Co. KG, Tuttlingen, Germany).^[^ ^40^ ^]^ Lecithine and cholesterol were used for liposome formulation. First, the lipids were dissolved in a mixture of chloroform and methanol (9:1; 100 mm) and mixed according to the respective compositions, as shown in Table S2 (Supporting Information). The organic solvents were evaporated under a nitrogen stream. Lipid films were dried in a vacuum chamber for 1 h at RT. Prior to dual centrifugation, 152 mg of Yttria stabilized zirconia ceramic beads (Sigmund Lindner GmbH, Warmensteinach, Germany) were added, and dual centrifugation was performed in three runs by addition of the required volumes of BSS without (Table S3, Supporting Information) or with MTX (Table S4, Supporting Information).

### Characterization of Phospholipid‐Based Liposomes

The average particle size, polydispersity index (PDI), and zeta potential of the liposomes were determined as the mean of three values by dynamic light scattering at room temperature using the automatic mode of a Zetasizer Ultra from Malvern. To determine particle size (z‐average) and PDI, liposomes were diluted with phosphate‐buffered saline (PBS) to obtain a lipid concentration of 0.01% (v/v). For zeta potential measurements, liposomes were mixed with 10% phosphate buffer solution (PBS) to a final concentration of 0.025% (v/v) of the respective liposomal formulation. The default settings of the automatic mode of the Zetasizer Ultra are presented in the Supporting Information.

### Cryo‐TEM

Liposomes loaded with MTX were applied to holey carbon coated grids (Lacey, Tedpella, USA) initially glow‐discharged during 45 sec at 20 mA in GloCube Plus (Quorum, UK). After 10 sec incubation at 10 °C and 90% humidity, the grids were blotted from the back side using Whatman grade 1 filter paper and vitrified in liquid ethane at −180 °C with a Leica GP2 plunger (Leica Microsystems, Vienna, Austria). Subsequently, the vitrified grids were transferred to a Talos 200 electron microscope (FEI, Hillsboro, OR, USA) using a Gatan 626 cryo‐holder (Gatan, Pleasanton, USA). Electron micrographs were acquired at an accelerating voltage of 200 kV using a low‐dose system (40 e‐ Å^−^^2^), while maintaining the sample at −175 °C. Defocus values ranged from −2 to −3 µm, and 25 images were captured on a 4 K × 4 K Ceta CMOS camera.

### In Vitro Cytotoxicity in ARPE‐19 Cell Culture

The human retinal pigment epithelial cell line ARPE‐19 (LGC Standards GmbH, Wesel, Germany) were cultivated in Dulbecco's modified Eagle's medium containing nutrient mixture F12 (1:1) (DMEM‐F12) (Gibco/BRL Life Technologies, Grand Island, NY) supplemented with 10% fetal bovine serum and a combination of penicillin and streptomycin (100 U mL^−1^, 100 µg mL^−1^) and incubated at 37 °C and 5% CO_2_. All supplements were purchased from Fisher Scientific, Schwerte, Germany. Cells were split upon reaching ≈90% confluence.

For the viability assay, cells at a density of 1 × 10^4^ and 3 × 10^4^ cells per well were seeded into a 96‐well microplate (Greiner Bio‐One International, Kremsmünster, Austria) and cultured for 24 h as described above. After incubation, liposomes were serially diluted in cell medium and added to the cells to reach final concentrations ranging from 0.005 to 1.25 mg mL^−1^. Cells were incubated with the liposomes as described above for another 24 h. PrestoBlue HS Cell Viability Reagent (Invitrogen, Life Technologies, Darmstadt, Germany) was added to the wells, and the plates were incubated at 37 °C and 5% CO_2_ for 2 h. Afterward, fluorescence was measured at 540 and 590 nm using an Infinite M200 PRO microplate reader (Tecan, Männedorf, Switzerland). Cell viability was calculated using the untreated control cells as a reference for 100% viable cells.

### Encapsulation Efficiency of MTX

The encapsulation efficiency of MTX was determined by reversed‐phase HPLC as described in a later paragraph. Details on the methodology are presented in the Supporting Information. In short, MTX concentration was measured prior and after non‐entrapped MTX was removed by size exclusion chromatography of the liposomes in accordance to Uhl et al.^[^ ^41^ ^]^

### Preparation of PLGA‐Implants

The mixture of PLGA‐implants was prepared according to Arrow et. al.^[^ ^12^ ^]^ In brief, 234 mg PLGA was dissolved in 60 mL acetone, and 1.1 mL (26.1 mg, 25 mg mL^−1^) MTX was added to receive a ratio of 10:1. The solvent was evaporated at room temperature overnight. For the moulding process, the substance was transferred into a glass syringe heated with a heat gun (HL1910E, STEINEL GmbH, Herzebrock, Germany) and ejected through the nozzle tip of the syringe with 6 bar using the oil injection mode of a megaTRON S3 (GEUDER AG, Heidelberg, Germany). The ejected long rod was transferred under laminar flow and cut into small parts with a weight of 4.00 mg each. The implants were stored in sterile tubes until further use.

### Scanning Electron Microscopy of PLGA‐Implants

The PLGA‐implants were glued to scanning electron microscopy stubs (Aluminium; Science Services, Munich, Germany) using carbon adhesive tape (Science Services, Munich, Germany) and sputter‐coated with 10 nm gold at 30 mA in an EM ACE 200 sputter coater (Leica Microsystems, Wetzlar, Germany). The samples were introduced into the focused ion beam – scanning electron microscope Crossbeam 550 (Zeiss, Oberkochen, Germany) and imaged at 1.5 kV with a secondary electron (Se2) detector.

### In Vitro Release Assay of PLGA‐Implants

The in vitro release model was performed with a syringe pump (Landgraf Laborsysteme HLL GmbH, Langenhagen, Germany) with a constant BSS flow of 2.5 µL min^−1^. To the syringe pump, a 5 mL syringe (BD Discardit II, Becton Dickinson S.A., Franklin Lakes, USA) filled with BSS was connected with a 3‐way tap (BD Connecta, Becton Dickinson S.A., Franklin Lakes, USA) as a posterior segment model. Through the stamp of the 5 mL syringe, a second 3‐way tap (BD Connecta, Becton Dickinson S.A., Franklin Lakes, USA) was connected with a cannula (BD Microlance 3, Becton Dickinson S.A., Franklin Lakes, USA) for sample collection.

The PLGA rod (4.0 mg) was placed in the 5 mL syringe filled with BSS, and the syringe pump was started. Every 24 h, 50 µL samples were taken and analyzed by reverse‐phase HPLC (RP‐HPLC).

### HPLC Analysis of Aqueous Humor Samples

The analysis of methotrexate was carried out with HPLC using an Agilent 1100 system with a Chromolith Performance RP‐18e 100 ×3 mm column (Merck KGaA, Darmstadt, Germany) as the stationary phase. The measurement was performed with a 5 min linear gradient starting at 100% water (+0.1% trifluoroacetic acid) to 100% acetonitrile (+0.1% trifluoroacetic acid), and the UV absorption at 258 nm was measured. As standard condition, 20 µL of each sample was injected and analyzed. For the determination of the concentration of methotrexate, a calibration curve between 0.06 and 62.5 µg mL^−1^ was created. The samples were measured in triplicates (y = 11,138x‐0,5303, R2 = 1). The HPLC diagrams for the sample with 125 µg mL^−1^ methotrexate and aqueous humor after 6 weeks exemplary, and the calibration curve is shown in (Figures S2 and S3 and Table S5, Supporting Information).

### Animals

Sixteen landrace pigs from a local farmer with an initial weight of 30 to 40 kg were used for this study (see Table 1). The experiment adhered to the ARVO Statement for the Use of Animals in Ophthalmic and Vision Research and was approved from the local ethics committee (Regierungspräsidium Karlsruhe, ethical approval number: 35–9185.81/G‐11/24). All animals used in the experimental laboratory were managed according to German laws for animal use and care, and according to the directives of the European Community Council (2010/63/EU) and ARRIVE guidelines.^[^ ^42^ ^]^ Pigs were housed in the Interfaculty Biomedical Research Facility, Heidelberg University, with water ad libitum and restricted food access. All procedures were performed on the right eye under sterile conditions using an operating microscope. Pigs were either used for pharmacokinetics or biocompatibility studies.

### Pharmacokinetics

The first group focused on analyzing drug release kinetics of either a dose of 400 µg of free MTX (*n* = 1), 400 µg of MTX encapsulated liposomes (*n* = 4) or PLGA implants loaded with 400 µg MTX (*n* = 2). Aqueous taps were performed at 24, 48, 72 h, 1, 2, 4, and 6 weeks after the IVI (Figure 2) to determine drug concentration using high‐performance liquid chromatography (HPLC). Animals were euthanized with an overdose of potassium chloride solution (7.45%) six weeks after injection.

### Biocompatibility

Nine animals received IVIs containing either unloaded (*n* = 3) or MTX‐loaded liposomes (*n* = 3) or SHAM‐injections of BSS (*n* = 3). Biocompatibility (see details below) was assessed 1‐, 2‐, 4‐, and 6‐weeks post‐IVI (Figure 2). After euthanasia, the eyes were enucleated for histological processing.

### Intravitreal Injection and Implantation of PLGA‐rods

For all procedures, animals were sedated with an intramuscular injection of Sedanol (WDT, Garbsen, Germany) (6 mg kg^−1^) and then anaesthetized with ketamine (CP Pharma, Burgdorf, Germany) (11 mg kg^−1^) and midazolam (Hameln Pharma, Hameln, Germany) (2 mg kg^−1^).^[^ ^43^ ^]^

The right eye of eight pigs received an intravitreal injection of either 400 µg MTX‐liposomes or unloaded liposomes. The injection volume was 0,075 mL with slight variation depending on the drug loading of different MTX‐liposome batches. The ocular surface was anesthetized with Conjuncain (Bausch+Lomb, Rochester, USA). After iodine disinfection, injections were placed at the pars plana with a 30G microsyringe. Topical antibiotics (Floxal, Bausch+Lomb, Rochester, USA) and corticosteroids (Isopto‐Max suspension, Novartis Pharma GmbH, Basel, Switzerland) were applied immediately after injection.

For the implantation of the PLGA implants, the eyes of two pigs received a solid 4 mg‐PLGA implant. Anesthesia was maintained throughout the procedure using intravenous propofol administration. The ocular surface was anesthetized with Conjuncain. The sclera was thinned, and hemostasis was achieved using diathermy. A 1.8 mm incision was made through the pars plana, and the rod was inserted into the vitreous cavity. The incision site was closed with 7‐0 Vicryl sutures, and topical antibiotics and corticosteroids were applied after the procedure. Video S1 demonstrates its implantation.

### Aqueous Taps

At 24, 28, 72 h, 1‐, 2‐, 4‐ and 6‐weeks post‐implantation, the eyes of the abovementioned 6 pigs received anterior chamber aqueous taps (Figure 2). Aqueous taps were performed using a 30G needle under direct visualization at the limbus. A volume of 50—100 µL of aqueous humor was aspirated. Video S2 demonstrates an aqueous tap procedure.

### In Vivo Biocompatibility Examination

Nine pigs underwent biocompatibility testing of both eyes after the injection of either unloaded liposomes (*n* = 3), MTX‐loaded liposomes (*n* = 3) or BSS (*n* = 3) after 1‐, 2‐, 4‐, and 6‐weeks postoperatively (Figure 2). First, intraocular pressure measurements were recorded using the iCare Tonovet Plus. After inducing mydriasis (cyclopentolate (0,5%), epinephrine (5%) and tropicamide (1%), fundus photographs of the posterior pole, including the optical nerve head, were acquired using the ClearView2 veterinary fundus camera (Optibrand, Fort Collins, USA). Subsequently, optical coherence tomography of the central retina was performed using the Spectralis Flex OCT (Heidelberg Engineering, Heidelberg, Germany). A 20° dense scan with a 30° infrared reflectance image was performed. The follow‐up function was used to examine the exact same location at every subsequent biocompatibility visit. Lastly, electroretinography was performed using the RETevet system (LKC Technologies, Gaithersburg, USA) and the Dog, Cat, Nonhuman Primate ISCEV 6 Step Light First test protocol. This test includes light‐ and dark‐adapted tests (15 min dark adaptation) to better assess cone and rod function. The ground electrode was placed centrally on the forehead. The reference electrode was placed 2.5 cm lateral to the outer canthus. The active electrode was placed directly on the cornea. Statistical comparison was performed at 6 weeks between the treated and non‐treated eye. The dark‐adapted dim flash test was omitted from statistical analyses as it showed a suboptimal signal‐to‐noise ratio in all eyes (control and experimental eyes) of all pigs.

### Histopathologic Assay of the Retina

Details on the methodology and used compounds are presented in the Supporting Information. For the eyes undergoing the biocompatibility testing, eyes were enucleated post‐mortem, fixed in 4% paraformaldehyde for 4 h, embedded in Optimal Cutting Temperature Compound (O.C.T) and cut into 10 µm thick sections using a cryostat. Hematoxylin and eosin staining was performed, and the sections were evaluated under a light microscope (Nikon Ni‐E research microscope, Nikon, Tokyo, Japan). Further, immunostainings for CD45 to assess leukocyte infiltration, Iba1 to assess microglia activation and GFAP to assess glial proliferation were conducted in both, left control eyes and eyes injected with MTX‐loaded liposomes.

### Statistical Analysis

Statistical analyses were performed using Prism 10 (GraphPad Inc, USA). Normality was assessed using the Kolmogorov‐Smirnov‐tests. Paired or unpaired *t*‐tests were applied as appropriate. Data is presented in mean ± SEM if not otherwise specified. *P*‐values of <0.05 were considered statistically significant.

## Conflict of Interest

MH, SW, GUA, and PU are shared inventors on a patent application for the examined MTX‐loaded phospholipid‐based liposomes.

## Supporting information



Supporting Information

Supplemental Video 1

Supplemental Video 2

## Data Availability

The data that support the findings of this study are available from the corresponding author upon reasonable request.
