# Supplementary material for: Methotrexate‐Loaded Liposomal Formulation Enables 6‐Week Sustained Intraocular Therapeutic Drug Release in a Porcine Model
Source: Adv Healthc Mater. 2025 Sep 25;15(1):e03230. doi: 10.1002/adhm.202503230 (PMC12790324; doi:10.1002/adhm.202503230)
Supplement: Supplementary file 1 — Supporting Information [file ADHM-15-0-s002.docx]

Supporting Information

Methotrexate-loaded liposomal formulation enables 6-week sustained intraocular therapeutic drug release in a porcine model

Maximilian Hammer, Lea Skrzypczyk, Sabrina Wohlfart, Simon William Pohl, Margarita Karaivanova, Jonathan Herth, Victor Aristide Augustin, Alexander Studier-Fischer, Tina Sackmann, Leon Kaulen, Anna Steyer, Walter Mier, David H Steel, Kanmin Xue, Gerd Uwe Auffarth, Philipp Uhl*

Full list of materials

1. Materials for liposomal formulations

Soy lecithin (SPC) was purchased from AppliChem GmbH (Darmstadt, Germany) and Methotrexate (25 mg/mL) (MTX) from medac GmbH, Wedel, Germany. Balanced sterile saline solution was obtained from BVI Medical (Heidelberg, Germany); Dulbecco’s phosphate buffered saline from Gibco® by life technologies^TM^ (Paisley, UK), while cholesterol, Triton™ X-100, chloroform and methanol were obtained from Sigma Aldrich (Taufkirchen, Germany).

1. Materials for PLGA-rods

Aceton was purchased at ZENTRALBEREICH Neuenheimer Feld, Heidelberg Germany, Methotrexate (25 mg/mL) was purchased at medac GmbH, Wedel, Germany and PLGA (EXPANSORB® DLG50) was purchased at SEQENS, Écully, France.

1. Materials for HPLC analysis

Water HPLC Gradient grade was purchased at Fisher Scientific (Loughborough, United Kingdom), acetonitrile CHROMASOLV^TM^ Gradient was purchased at Honeywell International Inc. (Morristown, USA) and trifluoroacetic acid was purchased at Biosolve B.V. (Valkenswaard, Netherlands)

1. Materials and preparation for histology

Porcine eyes were enucleated immediately post-mortem and fixed in 4% paraformaldehyde (ROTIHistofix 4%, Carl Roth GmbH + Co. KG, Karlsruhe, Germany) for 48 hours. Tissues were cryoprotected in a self-prepared ascending sucrose solution ((D+)-sucrose, >99.5%, Carl Roth GmbH + Co. KG, Karlsruhe, Germany) at concentrations of 10%, 20%, and 30% in PBS (80 g NaCl, 2.01 g KCl, 11.35 g Na₂HPO₄, 2.72 g KH₂PO₄ in 1L of water). Using a scalpel (blade 24, B. Braun SE, Melsungen, Germany), samples were prepared and embedded in Tissue Freezing Medium (LEICA Biosystems, Newcastle upon Tyne, United Kingdom) within cryomolds (Tissue-Tek Cryomold, 10 mm × 10 mm × 5 mm, SAKURA) and frozen at -24°C in a cryostat (LEICA CM 1850). Cryosections of 10 µm, 16 µm, and 30 µm thickness were cut (Microtome blades, C35 Type, FEATHER® Safety Razor Co. Ltd., Osaka, Japan) and mounted directly from the cryostat onto microscope slides (Superfrost Plus Adhesion Microscope Slides, white tab, Epredia Portsmouth, New Hampshire, USA), then air-dried for 12 hours at room temperature.

For H&E staining, slides were processed as following: rinsing with distilled water, staining with hematoxylin solution (acidic Mayer's hematoxylin solution, Carl Roth GmbH + Co. KG, Karlsruhe, Germany) for 6 minutes, bluing in running tap water for 15 minutes, rinsing with distilled water (2 minutes), staining with eosin G solution (0.5%, Carl Roth GmbH + Co. KG, Karlsruhe, Germany) with the addition of one drop of glacial acetic acid (99.8–100%, Bernd Kraft GmbH, Den Haag, Netherlands) for 3 minutes, followed by rinsing with tap water. Slides were dehydrated in an ascending series of Ethanol (70%, 96%, and 100%; ZENTRALBEREICH Neuenheimer Feld, Heidelberg, Germany), cleared in ROTIHistol (Carl Roth GmbH + Co. KG, Karlsruhe, Germany), mounted with ROTIHistokitt (Carl Roth GmbH + Co. KG, Karlsruhe, Germany), and covered with a coverslip (24 × 50 mm, borosilicate glass,

VWR International, Radnor, USA).

1. Immunostainings for GFAP, Iba1 and CD45

Porcine eyes were collected immediately after euthanasia and briefly stored in Balanced Salt Solution (BSS; BVI Medical, Heidelberg, Germany). The eye was opened at the equator, the anterior segment was removed, and the vitreous was carefully dissected. The posterior eyecup was then filled with isopentane (>99%; Carl-Roth, Karlsruhe, Germany), which had been pre-cooled in liquid nitrogen to ensure rapid preservation of tissue morphology. Subsequently, Tissue Freezing Medium (LEICA Biosystems, UK) was introduced into the eyecup, and the entire specimen was rapidly immersed in liquid nitrogen for immediate cryopreservation.

Cryosections with a thickness of 5–7 µm were prepared at −19 °C using a cryostat (LEICA CM1850, Germany) and mounted on Superfrost Plus microscope slides (Epredia, Germany). Sections were air-dried and fixed in pre-cooled acetone (>99.5%; Carl-Roth) at −10 °C for 10 minutes. Following an additional drying step, the sections were encircled with a hydrophobic barrier pen (Dako PAP Pen, Agilent Technologies, Santa Clara, USA) to enable targeted incubation.

Slices were pre-incubated with 10% horse serum (S-2000, Vector Laboratories, Burlingame, USA) in 1% BSA/PBS for 30 minutes in a humidified dark chamber. After removing the blocking solution, primary antibodies diluted in PBS were applied. These included anti-CD45 antibody (clones 2B11 + PD7/26, Dako, Agilent Technologies, Santa Clara, USA), anti-GFAP antibody (clone GF 12.24, PROGEN, Heidelberg, Germany), and anti-Iba1 antibody (HPA049234, Atlas Antibodies, Sigma-Aldrich, USA), each at a dilution of 1:100. Sections were incubated overnight at 4 °C.

On the following day, the sections were washed twice with PBS for five minutes each and incubated for 30 minutes at room temperature in the dark with DAPI (1:1000; D9542, Sigma-Aldrich, Merck, Germany), a Cy3-conjugated goat anti-mouse secondary antibody (1:200; Jackson ImmunoResearch, West Grove, USA), and Phalloidin (1:500; CytoPainter, ab176753, Abcam, Cambridge, UK). After two additional PBS washes (five minutes each, protected from light), sections were mounted using VecaMount (Vector Laboratories, USA) and sealed with borosilicate glass coverslips (24 × 50 mm, VWR).

Default settings of the automatic mode of the Zetasizer Ultra

The automatic mode of the Zetasizer Ultra from Malvern™ (Malvern Instruments Ltd., Worcestershire, United Kingdom) was used: number of measurements = 3; number of runs = 5 (size, PDI) or 50 (zeta potential); equilibration time = 60 s; refractive index solvent 1.330; refractive index polystyrene cuvette 1.590; viscosity = 0.8872 mPas; temperature = 25 °C; dielectric constant = 78.5 Fm^-1^; Smoluchowski equation.

Encapsulation Efficiency of MTX

After preparation, the liposomes were divided into two samples (20 µL each). The first sample was used to calculate the 100%-value after dissolving the liposomes by 20% Triton-X100 solution (1:5 v/v), while the other sample was purified from non-entrapped MTX by NAP™-5 size exclusion gel chromatography according to Uhl et al (2016)^[41]^. Fractions that contained visible MTX were pooled. Following dissolution with 20% Triton-X100 (1:10 v/v), the sample was analyzed by HPLC to calculate the relative amount of entrapped MTX by the following equation under consideration of different sample and fraction volumes:

$$E\left( \% \right)=\frac{\left[ \mathrm{AUC} \right]MTX part 2}{\left[ \mathrm{AUC} \right]MTX part 1}$$

Whereas [AUC] MTX part 2 is considered as the concentration of MTX in the liposomes after purification and [AUC] MTX part 1 is considered as the concentration of MTX in the liposomes before purification.

The drug loading DL (%) of the liposomes with MTX was calculated using the following equation:

$$DL\left( \% \right)=\frac{E\left( \% \right) x total amount MTX \left[ \mathrm{mg} \right]}{total amount lipid [mg]}$$

To determine the DL (%), the calculated encapsulation efficiency (%) was multiplied with the total amount of MTX (4.56 mg) and divided by the total mass of lipid (18.067 mg) used for the liposomal formulation.

**Table S1.** MTX-levels [µg/mL] in all animals undergoing pharmacokinetic testing

|  | MTX-Liposomes | | | | PLGA-Implants | | Free MTX |
| --- | --- | --- | --- | --- | --- | --- | --- |
| Days | Pig 1 | Pig 2 | Pig 3 | Pig 4 | Pig 1 | Pig 2 | Pig 1 |
| 1 | 2,9 | 2,7 | 1,7 | 2,9 | 5,0 | 4,3 | 0 |
| 2 | 3,7 | 1,8 | 2,1 | 2,8 | 4,4 | 4,6 | 5,1 |
| 3 | 4,2 | 2,3 | 2,8 | 4,7 | 4,8 | 5,0 | 4,9 |
| 8 | 4,9 | 4,4 | 3,4 | 7,7 | 6,2 | 5,7 | 1,8 |
| 13 | 7,0 | 5,7 | 3,7 | 9,8 | 5,3 | 5,5 | 0,3 |
| 28 | 5,9 | 5,7 | 3,2 | 9,6 | 6,9 | 4,6 | 0 |
| 42 | 6,7 | 6,0 | 6,4 | 10,9 | 6,7 | 6,4 | 0 |

Table S1 presents the MTX-concentrations (µg/mL) in the aqueous humor of all animals undergoing pharmacokinetic testing. The total injected dose for all animals was 400 µg and thus allows direct comparison of results. While no therapeutic concentrations were present in the animal injected with free MTX over the study period, both sustained drug release approaches allowed therapeutic levels over 6 weeks.

**Table S2.** Composition of the phospholipid-based liposomes.

|  | Ratio [mol-%] | Volume [µL] | Concentration [mM] | Molecular weight [g/mol] | Mass [mg] |
| --- | --- | --- | --- | --- | --- |
| Lecithin | 90 | 225 | 100 | 760 | 17,1 |
| Cholesterol | 10 | 25 | 100 | 386,7 | 0,97 |

**Table S3.** Characteristic settings of the dual centrifugation process (Zentrimix) for liposomal formulations with BSS.

| Run | Time [min] | Volume [µL] |
| --- | --- | --- |
| 1 | 15 | 27 |
| 2 | 5 | 90 |
| 3 | vortex | 133 |
|  |  |  |

Run 1 and 2 was performed with the Zentrimix (DC) at room temperature (20 °C) at 2.500 rpm.

**Table S4.** Characteristic settings of the dual centrifugation process for liposomal formulations with BSS and methotrexate.

| Run | Time [min] | Volume pure BSS [µL] | Volume Methotrexate in BSS [µL] |
| --- | --- | --- | --- |
| 1 | 15 | 0 | 27 |
| 2 | 5 | 71.4 | 18.6 |
| 3 | vortex | 133 | 0 |

Run 1 and 2 was performed with the Zentrimix (DC) at room temperature (20 °C) at 2.500 rpm.

**Figure S1.** Cryo-Transmission Electron Microscopy (cryo-TEM) of the methotrexate (MTX) liposomes after 24 hours of contact with silicone oil (Siluron 5000, Fluoron GmbH, Ulm, Germany) used as an intraocular endotamponade. The liposomes are still intact and similar in size as previously.

**Figure S2.** Exemplary HPLC plots.

(A) Exemplary HPLC curve for 125 µg/mL MTX

(B) 6-week time point of the aqueous humor sample of an animal treated with the MTX-loaded liposomes.

**Figure S3.** HPLC calibration curve.

With an R^2^ of >0.999, a reliable HPLC calibration curve was established.

**Table S5.** MTX concentration and corresponding AUC and standard deviation

| Concentration  [µg/mL] | AUC | SD | N |
| --- | --- | --- | --- |
| 62.5 | 695.13 | 1.04 | 3 |
| 31.25 | 348.60 | 0.58 | 3 |
| 15.625 | 173.89 | 0.14 | 3 |
| 7.8125 | 86.09 | 0.18 | 3 |
| 3.90625 | 41.40 | 0.74 | 3 |
| 1.953125 | 20.77 | 0.10 | 3 |
| 0.9765625 | 10.38 | 0.26 | 3 |
| 0.48828125 | 5.12 | 0.09 | 3 |
| 0.24414063 | 2.52 | 0.05 | 3 |
| 0.12207031 | 1.31 | 0.08 | 3 |
| 0.06103516 | 0.53 | 0.03 | 3 |
